# Structural and optical properties of ZnS thin films deposited by RF magnetron sputtering

**DOI:** 10.1186/1556-276X-7-26

**Published:** 2012-01-05

**Authors:** Dong Hyun Hwang, Jung Hoon Ahn, Kwun Nam Hui, Kwan San Hui, Young Guk Son

**Affiliations:** 1 School of Materials Science and Engineering, Pusan National University, Busan 609-735, South Korea; 2 Department of Systems Engineering and Engineering Management, City University of Hong Kong, Hong Kong, China

**Keywords:** ZnS film, RF magnetron sputtering, solar cell, Cd-free buffer layer

## Abstract

Zinc sulfide [ZnS] thin films were deposited on glass substrates using radio frequency magnetron sputtering. The substrate temperature was varied in the range of 100°C to 400°C. The structural and optical properties of ZnS thin films were characterized with X-ray diffraction [XRD], field emission scanning electron microscopy [FESEM], energy dispersive analysis of X-rays and UV-visible transmission spectra. The XRD analyses indicate that ZnS films have zinc blende structures with (111) preferential orientation, whereas the diffraction patterns sharpen with the increase in substrate temperatures. The FESEM data also reveal that the films have nano-size grains with a grain size of approximately 69 nm. The films grown at 350°C exhibit a relatively high transmittance of 80% in the visible region, with an energy band gap of 3.79 eV. These results show that ZnS films are suitable for use as the buffer layer of the Cu(In, Ga)Se_2 _solar cells.

## Background

Generally, Cu(In, Ga)Se_2 _[CIGS] solar cells are fabricated using a cadmium sulfide [CdS] buffer layer in order to protect the junction region from sputtering damage during subsequent n-type zinc oxide deposition and to modify the surface of p-type CIGS absorber [1]. CdS is the most promising buffer layer for thin film hetero-junction solar cells, and the highest conversion efficiencies have been achieved with the chemical bath-deposited CdS buffer layer in CIGS solar cells. The chemical bath deposition [CBD] technique, which is also known as solution growth or chemical deposition, has emerged as a rather efficient method for the deposition of metal chalcogenide thin films. This method is attractive largely because the technique possesses many advantages over other thin film deposition methods, such as low cost, low deposition temperature, and easy coating of large surfaces, making it appropriate for large area industrial applications. Over the years, many studies have been conducted to grow a buffer layer material (such as the CdS thin film) by this method [2-4]. However, the CdS layer fabricated by CBD causes serious environmental problems due to the large amount of cadmium-containing waste during the deposition process. Therefore, the development of a Cd-free buffer layer is one of the major objectives in the field of CIGS solar cells.

Today, zinc sulfide [ZnS] is considered one of the best materials for the CIGS solar cells among possible alternative buffer layers. In comparison with CdS, the advantages of ZnS include its non-toxic and environmentally safe handling as well as its ability to provide better lattice matching to CIGS absorbers having energy band gaps in the range of 1.3 to 1.5 eV compared with CdS and having a wider energy band gap compared with CdS, which transmits even higher energy photons and increases the light absorption in the absorber layer [5-7]. Several growth techniques, such as CBD [8], metal organic chemical vapor deposition [9], molecular beam epitaxy [10], and atomic layer epitaxy [11], have been applied to grow high quality ZnS films for device applications in electroluminescent displays and solar cells. Among these, radio frequency [RF] magnetron sputtering, a relatively cost-effective deposition technique compared with those listed above, has sufficient control over the stoichiometry and uniformity of the film employed to produce ZnS thin films [12-14].

In this study, we prepared ZnS thin films using RF magnetron sputtering. The influence of different substrate temperatures on the structural properties of the films has been investigated, and the optical properties of the films have also been analyzed.

## Methods

### Synthesis

ZnS films were deposited onto a Corning E2000 glass substrate (Corning Inc., Corning, NY, USA) by RF magnetron sputtering. The distance between the target and substrate was about 50 mm. The substrates were cleaned with acetone and isopropyl alcohol for 10 min each and then rinsed with deionized water before drying. After cleaning, the samples were immediately loaded into a chamber. Before deposition, the chamber was pumped down to a base pressure of 5.0 × 10^-5 ^Torr. A 50-mm diameter ZnS target (99.99%) was used for sputtering. In order to clean the surface of the target, pre-sputtering for 10 min was performed with an RF power of 120 W under pure argon gas while the substrate was covered with a shield. During ZnS film growth, argon gas with a flow rate of 55 sccm was fed through the mass flow controller into the chamber, and a working pressure of 3.0 × 10^-2 ^Torr was maintained. The deposition was continued for 20 min, and the sputter power was maintained at 120 W. The substrate temperature was varied from 100°C to 400°C. The typical sputtering conditions are listed in Table 1.

**Table 1:** Sputtering conditions of ZnS films

Parameter	Condition
Target	ZnS (99.99% pure)
Substrate	Corning E2000 glass
RF power	120 W
Sputtering gas	Pure argon (55 sccm)
Deposition time	20 min
Sputtering pressure	3 × 10^-2 ^Torr
Substrate temperature	100**°C**, 200**°C**, 250**°C**, 300**°C**, 350**°C**, 400**°C**
Target to substrate distance	50 mm

### Characterizations

The crystalline phase of the films was studied with an X-ray diffractometer [XRD] (Bruker D8 Advance; Bruker, Billerica, MA, USA) using Cu Kα radiation (*λ *= 0.15406 nm) operated at 40 kV and 40 mA. The surface morphology and grain size of the films were determined by FESEM (Hitachi S-4800; Hitachi, Ltd., Tokyo, Japan). The thickness of the films was estimated using the cross-sectional FESEM image. The composition of the films on glass substrates was investigated by energy dispersive analysis of X-ray [EDAX] (Horiba 7593-H; Horiba, Ltd., Kyoto, Japan). The optical properties of the films were characterized by a UV-Visible spectrometer (Shimadzu UV-1800; Shimadzu Corp., Kyoto, Japan) with a wavelength range from 200 to 1,100 nm.

## Results and discussion

### Structural properties

**Figure 1:**
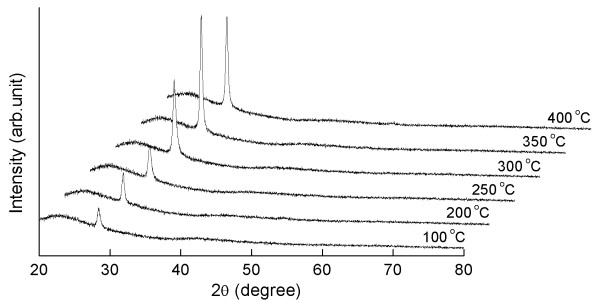
**XRD patterns of ZnS films grown at various substrate temperatures from 100°C to 400°C**.

Figure 1 shows XRD patterns of ZnS thin films formed by sputtering at different substrate temperatures ranging from 100°C to 400°C. One peak (2*θ *≈ 28.50°) was significantly observed for every film in the diffraction angle (2*θ*) range from 20° to 80°. This indicated that the films were single crystalline structures with a preferential orientation, and that the planes were parallel to the substrate surface. All the films grown at various substrate temperatures only had an (111) plane and exhibited a zinc blende structure. As the substrate temperature increased to 350°C, the intensity of the peaks corresponding to the cubic phase also increased drastically. Further increments in the substrate temperature up to 400°C resulted in a slight reduction in the intensity of the cubic phase. The highest peak value of the XRD measurement came from the ZnS film grown at 350°C, indicating that the film had the best preferred orientation structures.

In order to obtain more structural information, the mean crystallite sizes (*D*) of the films are calculated using Scherrer formula [15]:

(1) D= 0.9λ∕(βcosθ),

where *λ *is the X-ray wavelength (0.15406 nm), and *β *is the full width at half maximum [FWHM] of the film diffraction peak at 2*θ*, where *θ *is the Bragg diffraction angle. The FWHM value decreased from 0.384° to 0.141° as the deposition temperatures increased from 100°C to 350°C. However, the FWHM value of the film prepared at 400°C increased to 0.154°, indicating the deterioration of the crystallinity of the films. The mean crystallite sizes of the films were about 22.3, 25.5, 29.8, 43.1, 60.8, and 55.6 nm for samples deposited at 100°C, 200°C, 250°C, 300°C, 350°C, and 400°C, respectively. These results were probably due to the crystallinity of the films being improved and the crystallite sizes becoming larger as the substrate temperatures increased. Crystallinity is highly related to FWHM value. Valenzuela and Russer reported that the FWHM of an XRD peak is reliant on the crystallite size and the lattice strain caused by the defect and/or dislocations [16]. The average particle sizes and FWHM values of the films are summarized in Table 2.

**Table 2:** Estimated FWHM and crystallite sizes of ZnS films grown at various substrate temperatures

Substrate temperature (°C)	FWHM values (degrees)	Crystallite size by XRD (nm)	Grain size by FESEM (nm)
100	0.384	22.3	27.2
200	0.314	25.5	30.1
250	0.288	29.8	36.5
300	0.199	43.1	50.3
350	0.141	60.8	69.4
400	0.154	55.6	66.2

Table 3 shows the variation of Zn and S chemical compositions in ZnS films under different substrate temperatures analyzed by EDAX, using an acceleration voltage of 15 kV. The size of the investigated area was above 100 × 100 μm. All samples prepared at various substrate temperatures were non-stoichiometric, and Zn had more content than S except in the films grown at 350°C. The Zn/S ratio decreased slowly as the substrate temperatures were elevated. When the temperature reached to 350°C, the ZnS film showed nearly equal counts for Zn and S, indicating that the film was stoichiometric and that the average Zn/S ratio for this film was about 0.99, corresponding to the smallest FWHM value in Table 2. From these results, we can infer that the film composition evolved with the growth temperatures. However, as the substrate temperature increased to 400°C, the Zn/S ratio of the films also increased to 1.04. This increase can be attributed to the re-evaporation of sulfide from the film surface.

**Table 3:** Chemical composition of ZnS films deposited at various substrate temperatures

Substrate temperature (°C)	Zn (atomic %)	S (atomic %)	Zn/S ratio
100	54.32	45.68	1.19
200	53.87	46.13	1.17
250	53.75	46.25	1.16
300	52.95	47.05	1.13
350	49.91	50.09	0.99
400	50.97	49.03	1.04

**Figure 2:**
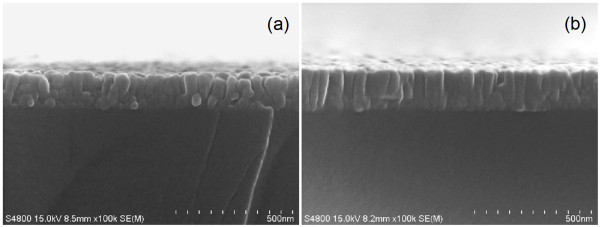
**Cross-sectional FESEM images of ZnS films grown at different substrate temperatures**. (**a**) 100**°C **and (**b**) 350**°C**.

The film thickness of ZnS films prepared at different substrate temperatures was estimated using cross-sectional FESEM images shown in Figure 2. From these images, the film thickness slightly increased from 145 nm to 165 nm as the growth temperature increased to 350°C. This can be deduced from the XRD results that the crystallite size reaches a maximum at 350°C. The average film thickness of all samples was about 155 nm.

**Figure 3:**
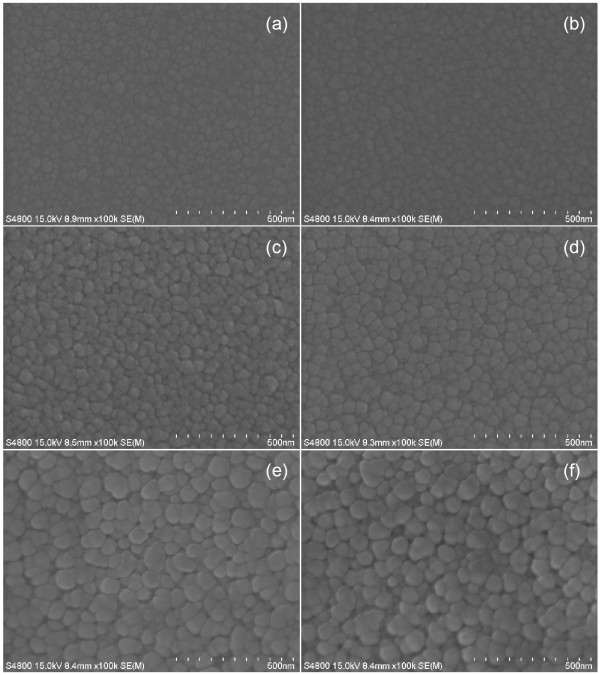
**FESEM images of ZnS films grown at various substrate temperatures**. (**a**) 100**°C**, (**b**) 200**°C**, (**c**) 250**°C**, (**d**) 300**°C**, (**e**) 350**°C**, and (**f**) 400**°C**.

The influence of the substrate temperatures on the surface morphology of the films was investigated using the FESEM images as shown in Figure 3. The morphology of the films was found to be continuous and dense. The average particle sizes varied in the range of 27.2 to 69.4 nm. The crystallinity of the films improved, and crystalline size along the surface became larger as the deposition temperatures increased. This improvement is due to the distant migration of the sputtered atoms, thus forming a denser film with larger grains and lower defects.

### Optical properties

**Figure 4:**
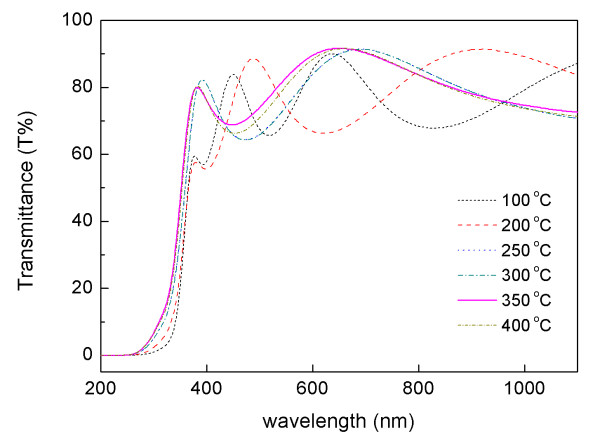
**Transmittance vs. wavelength spectra of ZnS films grown at various substrate temperatures**.

The optical transmittance spectra in the wavelength range of 200 to 1,100 nm of ZnS films deposited at different substrate temperatures are shown in Figure 4. The films deposited at 100°C and 200°C have relatively lower transparency, and small shoulders were observed in the absorption line. However, the images still exhibit an average transmittance of above 70%. The film formed at 350°C was relatively higher than the spectral transmittance for the other films prepared at other growth temperatures; moreover, the average transmittance in the visible region was above 80%. The reason for this is that the film was fabricated with a high degree of crystallinity, as indicated in Figure 1. The optical transmittance was also increased along with the increase in substrate temperature. The shift in the absorption line towards a higher energy side can also be attributed to the increase in substrate temperature [6].

**Figure 5:**
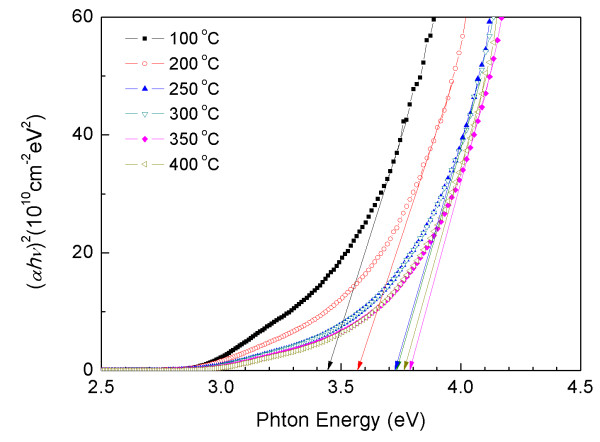
**Plot of (*α*hν)^2 ^vs. photon energy (hν) for ZnS films grown at various substrate temperatures**.

Figure 5 shows the plot of (*α*hν)^2 ^versus hν, where *α *is the optical absorption coefficient, and hν is the energy of the incident photon. The optical band gap (*E*_g_) is calculated from the following expression by assuming a direct transition between valance and conduction bands [17]:

(2) $$\alpha \text{h}\nu =D{\left(\text{h}\nu -E_{\text{g}}\right)}^{\text{1}∕\text{2}},$$

where *D *is a constant, and *E*_g _is estimated by extrapolating the straight-line portion of the spectrum to a zero absorption coefficient value. The optical band gap of the film deposited at 100°C was 3.45 eV. As the growth temperature increased from 200°C to 350°C, the optical band gap red-shifted from 3.57 to 3.79 eV. The band gaps between the film formed at 250°C (*E*_g _= 3.72 eV) and those grown at 300°C (*E*_g _= 3.73 eV) were slightly changed along with the deposition temperatures. The band gap of the film also decreased with the temperature up to 400°C (*E*_g _= 3.76 eV). These results indicate that an increase in the substrate temperature improves the band gap energy of the films.

## Conclusions

ZnS thin films have been successfully grown on glass substrates using RF magnetron sputtering at various substrate temperatures ranging from 100°C to 400°C. The influence of substrate temperature on the structural and optical properties of ZnS films prepared in the experiment has been characterized. The XRD measurements reveal that the films deposited at 350°C have a strongly (111) preferred orientation and are parallel to the substrate surface. The smallest FWHM value of 0.141° has also been observed for these films, indicating that the crystallinity of the films can be improved by increasing the substrate temperatures. All of the ZnS films deposited at different substrate temperatures are Zn-rich and S-deficient in terms of EDAX results. However, the Zn/S ratio of the films formed at 350°C is 0.99, indicating an ideal stoichiometric proportion of ZnS. The surface morphology studied by FESEM has shown that the grain sizes of ZnS films are influenced by the substrate temperatures. The films formed at 350°C exhibited good optical properties with a relatively high transmittance of 80% in the visible region, and the energy band gap is estimated to be 3.79 eV.

## Competing interests

The authors declare that they have no competing interests.

## Authors' contributions

DHH designed and carried out the experiments and wrote the first draft of the manuscript. JHA analyzed the properties and helped draft the manuscript. KNH and KSH detailed the original idea and modified the first draft of manuscript. YGS finalized the manuscript and supervised the work. All authors read and approved the final manuscript.
